# BMI, Sleep Architecture, and Glucose Metabolism: Insights From the Baependi Heart Study

**DOI:** 10.1002/oby.24359

**Published:** 2025-07-30

**Authors:** Carolina Mendes Pessoa, Tâmara P. Taporoski, Felipe Beijamini, Shaina J. Alexandria, Jose E. Krieger, Malcolm von Schantz, Alexandre C. Pereira, Kristen L. Knutson

**Affiliations:** ^1^ Feinberg School of Medicine Northwestern University Chicago Illinois USA; ^2^ Center for Population and Development Studies, Harvard T.H. Chan School of Public Health Cambridge Massachusetts USA; ^3^ Federal University of Fronteira Sul Realeza Paraná Brazil; ^4^ Incor University of São Paulo School of Medicine São Paulo São Paulo Brazil; ^5^ Faculty of Health and Life Sciences Northumbria University Newcastle Upon Tyne UK; ^6^ Brigham and Women's Hospital Harvard Medical School Boston Massachusetts USA

## Abstract

**Objective:**

To examine whether (1) sleep architecture is associated with BMI groups in the absence of sleep apnea and (2) BMI group modified associations between sleep architecture and markers of glucose metabolism.

**Methods:**

The Baependi Heart Study (BHS) is a family‐based observational study of adults that assessed sleep using at‐home polysomnography (PSG) and collected anthropometric and fasting blood measures. BMI was classified into: 18.5 to < 25, 25 to < 30, and ≥ 30 kg/m^2^. People with moderate–severe sleep apnea and taking diabetes‐related medication were excluded. Cross‐sectional associations were examined (*n* = 1014).

**Results:**

Individuals with BMI ≥ 30 kg/m^2^ had less REM sleep (−7.8 min, *p* = 0.003) and the groups with BMI 25 to < 30 kg/m^2^ and ≥ 30 kg/m^2^ had higher apnea‐hypopnea index than individuals with BMI 18.5 to < 25 kg/m^2^ (by 0.8 and 1.4 events per hour, respectively, *p* < =0.002). Wake after sleep onset (WASO) was associated with higher fasting glucose levels in participants with BMI ≥ 30 kg/m^2^ only; 10 min more WASO was associated with ~1% higher fasting glucose (*p* = 0.002).

**Conclusions:**

After exclusion of participants with moderate–severe sleep apnea, there was no difference in non‐REM sleep and only a small difference in REM sleep between BMI groups, suggesting that BMI does not substantially impair sleep unless sleep apnea is present.

**Study Importance**

- What is already known?
  ○ Obesity is linked to altered sleep patterns and impaired glucose metabolism.
  ○ Sleep apnea often confounds studies on sleep architecture in individuals with obesity.
- What does this study add?
  ○ After excluding moderate to severe sleep apnea, only minimal differences in sleep architecture were observed between BMI groups.
  ○ Sleep architecture was not associated with markers of glucose metabolism in participants whose BMI was < 30 kg/m^2^. In those with BMI > 30 kg/m^2^, greater wake after sleep onset (WASO) was associated with slightly elevated fasting glucose.
- How might these results change the direction of research or the focus of clinical practice?
  ○ Impaired sleep health among people with BMI > 25 kg/m^2^ may largely be a result of sleep apnea.

## 1 Introduction

Obesity is a major risk factor for obstructive sleep apnea (OSA), which is a breathing‐related sleep disorder [1] associated with cardiometabolic diseases [2, 3]. Inadequate sleep duration and quality, even without OSA, have also been associated with increased appetite, energy intake, and diabetes [4, 5, 6, 7]. However, it remains unknown whether, in the absence of moderate–severe OSA, sleep architecture varies among those with higher BMI, which is the primary objective of this analysis. Our secondary objective was to examine associations between sleep architecture and markers of glucose metabolism and to determine whether BMI modified this association.

## 2 Methods

### 2.1 Sample

Participants are from the Baependi Heart Study (BHS), a Brazilian family‐based cohort study of adults aged 18–91 years living in a typical rural town [8]. A cross‐sectional ancillary study to the BHS measured sleep using polysomnography (PSG), measured anthropometrics, and collected fasting blood samples between 2019 and 2023. All BHS participants were eligible for the ancillary study and all participants provided written informed consent. The protocol was approved by the ethics committee of the Hospital das Clínicas, University of São Paulo, Brazil.

### 2.2 Measurements

#### 2.2.1 Polysomnography (PSG)

Sleep architecture refers to amount and proportion of sleep stages, including wakefulness, rapid eye movement (REM) sleep, and non‐REM (further divided into stages N1, N2, and N3). Trained research staff visited the participants' homes on one night to set up the PSG (Trackit, Nihon Kohden, Tokyo, Japan), which included electroencephalography (EEG), electrooculography (EOG), electromyography (EMG), electrocardiography (ECG), oronasal airflow, respiratory effort from thoracic and abdominal piezoelectric belts, and pulse oximetry. Each 30‐s epoch was scored as Wake, N1, N2, N3, or REM following standard criteria [9] by an experienced rater blinded to the participant demographics. An apnea was scored when oxygen saturation dropped by ≥ 90% for ≥ 10 s and hypopneas was scored when there was a ≥ 10‐s drop of ≥ 30% in the respiratory effort and linked to either a 3% desaturation or an arousal [9]. We excluded participants with an apnea‐hypopnea index (AHI) ≥ 15 events per hour. Recordings with < 4 h of sleep were considered invalid.

#### 2.2.2 Anthropometrics

A stadiometer was used to measure height (cm) and a scale measured weight (kg), and both were assessed without shoes. Body mass index (BMI, kg/m^2^) was calculated and participants were grouped into: BMI 18.5 to < 25 kg/m^2^ (not overweight), BMI 25 to < 30 kg/m^2^ (overweight), and BMI ≥ 30 kg/m^2^ (obesity) [10].

#### 2.2.3 Markers of Glucose Metabolism

A fasting blood sample was collected in the morning after the PSG, on which hemoglobin A1c (HbA1c), glucose, and insulin levels were measured. The homeostatic model of insulin resistance (HOMA‐IR) was calculated as (glucose, mg/dL x insulin, uIU/mL)/405 [11]. Participants reporting taking diabetes‐related medication were excluded.

### 2.3 Statistical Analysis

The final sample size was 1045 participants. (Figure S1 provides a flowchart detailing exclusions.) Linear regression models compared PSG measures among BMI groups (BMI 18.5 to < 25 kg/m^2^ was referent) adjusted for age, gender, and AHI. Linear regression was also used to examine associations between the PSG measures and HbA1c, fasting glucose, and HOMA‐IR in unadjusted models and models adjusted for age, gender, and BMI group. The three glucose metabolism variables were transformed using the natural log (ln) due to skewness. Finally, we tested whether associations varied among the BMI groups by introducing an interaction term to the model. If the interaction term was significant (*p* < 0.05), models were stratified by BMI group. All analyses were performed using Stata SE (v 14, StataCorp LLC).

## 3 Results

Table 1 describes the sample overall and by BMI group. Average age was 46 years, and mean age was higher in the groups with BMI ≥ 25 and > 30 kg/m^2^. Women constituted 64.2% of the sample, and there was a higher proportion of women in the BMI > 30 kg/m^2^ group (72.8%). Figure S2 provides distribution of BMI in this sample.

**TABLE 1:** Description of full sample and by BMI group (all AHI < 15 events per hour).

Variable	Full sample	BMI 18.5 < 25 kg/m^2^	BMI 25 to < 30 kg/m^2^	BMI ≥ 30 kg/m^2^
*N*	1014	443	376	195
	Mean (SD) or *N* (%)	Mean (SD) or *N* (%)	Mean (SD) or *N* (%)	Mean (SD) or *N* (%)
Age (years)	46.3 (13.7)	45.3 (14.4)	47.0 (12.6)	47.1 (14.0)
Women, *n* (%)	651 (64.2%)	276 (62.3%)	233 (62.0%)	**142 (72.8%)** ^‡^
BMI (kg/m^2^)	26.4 (4.6)	22.5 (1.8)	**27.2 (1.4)** ^‡^	**33.7 (3.5)** ^‡^
Sleep measures				
TST (hours)	6.6 (1.0)	6.7 (1.0)	6.7 (1.1)	6.5 (1.1)
N2 (min)	225.8 (51.7)	224.3 (50.4)	227.5 (51.3)	226.2 (55.7)
N3 (min)	48.5 (36.1)	49.5 (37.2)	46.7 (33.6)	49.7 (38.2)
REM (min)	81.3 (31.4)	83.2 (30.9)	82.6 (31.8)	**74.7 (31.2)** ^**‡**^
N2 (%)	56.6 (9.5)	56.2 (9.6)	56.6 (8.9)	57.7 (10.2)
N3 (%)	12.2 (9.0)	12.3 (9.2)	11.8 (8.5)	12.7 (9.6)
REM (%)	20.2 (6.7)	20.6 (6.5)	20.3 (6.6)	**19.0 (7.1)** ^**‡**^
WASO (min)	54.6 (41.3)	52.2 (39.5)	56.1 (42.3)	56.9 (43.2)
AHI (events/hour)	3.8 (4.1)	3.1 (3.8)	**4.1 (4.1)** ^**‡**^	**4.6 (4.4)** ^**‡**^
Metabolic measures				
Glucose (mg/dL)	82.7 (13.2)	80.5 (10.7)	**82.9 (13.7)** ^**‡**^	**87.1 (15.8)** ^**‡**^
Insulin (µIU/mL)	10.1 (6.6)	7.6 (3.8)	**10.3 (6.3)** ^**‡**^	**15.4 (8.7)** ^**‡**^
HOMA‐IR	2.1 (1.6)	1.5 (0.8)	**2.1 (1.6)** ^**‡**^	**3.3 (2.0)** ^**‡**^
Hba1c (%)	5.5 (0.6)	5.3 (0.4)	**5.5 (0.8)** ^**‡**^	**5.6 (0.6)** ^**‡**^

^**‡**^ Unadjusted *p* value < 0.05 compared to BMI < 25.

Many sleep measures were similar across BMI groups in unadjusted (Table 1) and adjusted analyses (Table 2). Specifically, there were no differences in TST, amount or percentage of N2 or N3, or duration of wake after sleep onset (WASO). Participants with BMI ≥ 30 kg/m^2^ had less REM sleep (approximately 8 min less on average, or 1.6% of sleep period less). Individuals with BMI between 25 and < 30 kg/m^2^ and BMI ≥ 30 kg/m^2^ had a significantly higher mean AHI. Figure S2 presents distributions of TST and REM by BMI group. We confirmed that sleep architecture varied if OSA was present (Table S1); participants with moderate–severe OSA had less N3 and more WASO.

**TABLE 2:** Adjusted comparisons of PSG sleep measures by BMI group^a^.

Variable	BMI < 25	BMI 25 to < 30	BMI ≥ 30
	Mean (SD)	B (95% CI)	B (95% CI)
TST (hours)	Referent	0.06 (−0.08, 0.20) *p* = 0.43	−0.11 (−0.28, 0.07) *p* = 0.22
N2 (min)	Referent	3.4 (−3.8, 10.5) *p* = 0.36	2.8 (−6.0, 11.7) *p* = 0.53
N3 (min)	Referent	−0.6 (−5.2, 4.1) *p* = 0.81	1.6 (−4.1, 7.3) *p* = 0.58
REM (min)	Referent	0.1 (−4.0, 4.3) *p* = 0.95	**−7.8 (−12.9, −2.6)** ^**‡**^ ***p* = 0.003**
N2 (%)	Referent	0.3 (−1.0, 1.5) *p* = 0.68	1.5 (−0.04, 3.1) *p* = 0.06
N3 (%)	Referent	−0.04 (−1.2, 1.1) *p* = 0.94	0.7 (−0.8, 2.1) *p* = 0.35
REM (%)	Referent	−0.2 (−1.1, 0.7) *p* = 0.64	**−1.6 (−2.7, −0.5)** ^**‡**^ ***p* = 0.006**
WASO (min)	Referent	1.6 (−3.8, 6.9) *p* = 0.57	2.4 (−4.2, 9.0) *p* = 0.47
AHI (events/hour)	Referent	**0.8 (0.3, 1.3)** ^**‡**^ ***p* = 0.002**	**1.4 (0.8, 2.0)** ^**‡**^ ***p* < 0.001**

Abbreviation: B, unstandardized regression coefficient.

^a^ Adjusted for age, gender and AHI (model predicting AHI was only adjusted for age and gender).

^‡^ Adjusted *p* < 0.05 compared to BMI < 25.

Although there were several significant unadjusted associations between sleep architecture and the metabolic measures, only one remained significant after adjusting for covariates (Table 3): Ten minutes more WASO was associated with 1% higher HOMA‐IR. We also observed only one significant interaction term, which was in the model examining the association between WASO and fasting glucose. Therefore, we stratified these analyses by BMI group. There was no association between WASO and In(glucose) for those with BMI < 25 kg/m^2^ (*B* = 0.0005 per 10 min, 95% CI −0.003, 0.004) or those with BMI 25 to < 30 kg/m^2^ (*B* = −0.001 per 10 min, 95% CI −0.005, 0.002). For those with BMI ≥ 30 kg/m^2^, greater WASO was associated with higher fasting glucose levels (*B* = 0.008, 95% CI 0.003, 0.014), which indicated that 10 min more WASO was associated with approximately 1% higher glucose.

**TABLE 3:** Regression models comparing sleep stages to metabolic outcomes.

	ln(HbA1c)		ln(glucose)		ln(HOMA)	
	1041		1045		1042	
Variable	Unadjusted	Adjusted^a^	Unadjusted	Adjusted^a^	Unadjusted	Adjusted^a^
*N*
B (95% CI)	B (95% CI)	B (95% CI)	B (95% CI)	B (95% CI)	B (95% CI)
TST (hours)	**−0.006** ^‡^ **(−0.01, −0.0001)** ***p* = 0.045**	0.001 (−0.004, 0.006) *p* = 0.76	−0.001 (−0.011, 0.006) *p* = 0.75	0.005 (−0.004, 0.013) *p* = 0.26	−0.015 (−0.05, 0.02) *p* = 0.40	−0.011 (−0.043, 0.021) *p* = 0.49
N2 (per 10 min)	−0.0001 (−0.001, 0.001) *p* = 0.88	−0.0003 (−0.001, 0.001) *p* = 0.64	0.001 (−0.0004, 0.003) *p* = 0.16	0.001 (−0.001, 0.002) *p* = 0.32	−0.001 (−0.01, 0.006) *p* = 0.72	−0.002 (−0.01, 0.004) *p* = 0.56
N3 (per 10 min)	**−0.002** ^‡^ **(−0.003, −0.0001)** ***p* = 0.037**	0.001 (−0.001, 0.003) *p* = 0.18	**−0.004** ^‡^ **(−0.01, −0.001)** ***p* = 0.002**	−0.001 (−0.003, 0.002) *p* = 0.48	0.002 (−0.01, 0.01) *p* = 0.76	−0.003 (−0.01, 0.01) *p* = 0.49
REM (per 10 min)	**−0.003** ^‡^ **(−0.01, −0.001)** ***p* = 0.001**	−0.00001 (−0.002, 0.002) *p* = 0.99	−0.001 (−0.004, 0.002) *p* = 0.42	0.002 (−0.001, 0.005) *p* = 0.13	−0.0001 (−0.01, 0.01) *p* = 0.99	0.005 (−0.005, 0.02) *p* = 0.33
WASO (per 10 min)	**0.003** ^‡^ **(0.002, 0.005)** ***p* < 0.001**	0.001 (−0.001, 0.002) *p* = 0.44	**0.004** ^‡^ **(0.002, 0.006)** ***p* < 0.001**	0.002 (−0.001, 0.004) *p* = 0.16	0.007 (−0.002, 0.02) *p* = 0.15	**0.01** ^‡^ **(0.0001, 0.02)** ***p* = 0.046**
AHI (events/hour)	**0.004** ^‡^ **(0.003, 0.006)** ***p* < 0.001**	0.001 (−0.0005, 0.002) *p* = 0.18	**0.006** ^‡^ **(0.003, 0.008)** ***p* < 0.001**	0.002 (−0.0001, 0.004) *p* = 0.06	**0.01** ^‡^ **(0.001, 0.020)** ***p* = 0.031**	0.005 (−0.003, 0.01) *p* = 0.22

Abbreviations: B, unstandardized regression coefficient; ln, natural log transformation.

^a^ Adjusted for BMI group, age, and gender.

^‡^ *p* value < 0.05.

## 4 Discussion

We observed few differences in PSG sleep characteristics between BMI groups in the absence of moderate–severe sleep apnea, suggesting that having BMI > 25 kg/m^2^ does not in itself have a large, negative impact on sleep architecture. The primary difference was 8 min less REM sleep in participants with BMI > 30 kg/m^2^; however, the clinical significance of 8 min less REM is unknown. We also observed few associations between sleep architecture and markers of glucose metabolism; only greater WASO wake was weakly associated with insulin resistance (full sample) and glucose levels (those with BMI > 30 kg/m^2^ only). Therefore, these results suggest little impact of BMI ≥ 25 kg/m^2^ on sleep architecture beyond sleep apnea and minimal associations with glucose regulation.

The link between higher BMI and higher prevalence of sleep apnea is well established [1, 12, 13], but other PSG characteristics, such as sleep architecture, in relation to obesity status has only been examined in two studies. The Sleep Heart Health Study (SHHS) was a large observational study with in‐home PSG, and it reported greater WASO among the participants with obesity [14]; however, this study included people with sleep apnea. The SHHS did not report sleep stage data, so it is unknown if there were differences in N2, N3 or REM. A study of 400 adult women (aged 20–69 years) in Sweden recorded in‐home PSG and oversampled snorers [15]. They found that women with central obesity (i.e., waist circumference ≥ 88 cm) had less N3, less REM sleep, and higher AHI in unadjusted comparisons. When adjusting for AHI, they found a significant negative association between waist circumference and REM minutes but no association with N3 minutes, results which are consistent with our study. Together, these findings suggest less REM among individuals with greater BMI, but whether there are clinical implications of this remain to be determined.

We observed no significant associations between the PSG sleep measures and markers of glucose metabolism after adjusting for age, gender, and BMI group. Prior experimental studies that suppressed stage N3 sleep did observe impairments in glucose metabolism [16, 17, 18]; however, these studies were in young, healthy individuals who were not overweight. Most prior research that examined sleep and diabetes risk measured sleep via self‐report or wrist actigraphy; few studies have examined sleep architecture using PSG. The SHHS found that in those without sleep apnea, people with diabetes had low sleep efficiency and lower REM percentage compared to those without diabetes, but they did not differ in percentage of N3 [19]. Our large observational study, which included a wide range of ages and BMI, did not reveal associations between sleep stages, such as N3, and the markers of glucose metabolism in cross section. When examining whether associations were modified by BMI group, we found that more WASO was associated with higher fasting glucose only in participants with BMI > 30 kg/m^2^, but the effect size was small. No other associations varied by BMI group. Longitudinal studies would provide deeper insights into the bidirectional influences between sleep and diabetes among people at different levels of BMI.

Strengths of this study include the large sample size and objective measurement of sleep using PSG conducted in participants' homes, which enhances ecological validity. However, the study's generalizability outside of Brazil or in urban centers may be limited by confounders such as lifestyle. Further, behavioral factors that may vary by BMI and be associated with sleep architecture are not available in these data (e.g., diet quality, physical activity). The cross‐sectional design restricts the ability to infer causality. A single PSG may not account for changes in sleep that could be seen with serial studies. Nonetheless, these cross‐sectional data from a large cohort with a range of ages and BMI suggest minimal differences in sleep architecture by BMI and limited associations with glucose regulation once moderate–severe sleep apnea is excluded.

## Conflicts of Interest

The authors declare no conflicts of interest.

## Supporting information


**Figure S1.** Flowchart detailing exclusion criteria and sample size.
**Figure S2.** Histogram of the distribution of BMI in this sample.
**Figure S3.** Histograms for the distribution of TST and REM by BMI group in this sample.
**Table S1.** Adjusted comparisons of PSG sleep measures by BMI and AHI group.

## Data Availability

The data that support the findings of this study are available from the corresponding author upon reasonable request.

## References

[1] S. C. Veasey and I. M. Rosen , “Obstructive Sleep Apnea in Adults,” New England Journal of Medicine 380 (2019): 1442–1449. 30970189 10.1056/NEJMcp1816152

[2] S. Pamidi , R. S. Aronsohn , and E. Tasali , “Obstructive Sleep Apnea: Role in the Risk and Severity of Diabetes,” Best Practice & Research. Clinical Endocrinology & Metabolism 24 (2010): 703–715. 21112020 10.1016/j.beem.2010.08.009 PMC2994098

[3] S. Reutrakul and B. Mokhlesi , “Obstructive Sleep Apnea and Diabetes: A State of the Art Review,” Chest 152 (2017): 1070–1086. 28527878 10.1016/j.chest.2017.05.009 PMC5812754

[4] K. Spiegel , E. Tasali , P. Penev , and E. Van Cauter , “Sleep Curtailment in Healthy Young Men Is Associated With Decreased Leptin Levels, Elevated Ghrelin Levels, and Increased Hunger and Appetite,” Annals of Internal Medicine 141 (2004): 846–850. 15583226 10.7326/0003-4819-141-11-200412070-00008

[5] M.‐P. St‐Onge , A. L. Roberts , J. Chen , et al., “Short Sleep Duration Increases Energy Intakes but Does Not Change Energy Expenditure in Normal‐Weight Individuals,” American Journal of Clinical Nutrition 94 (2011): 410–416. 21715510 10.3945/ajcn.111.013904 PMC3142720

[6] F. P. Cappuccio , F. M. Taggart , N. B. Kandala , et al., “Meta‐Analysis of Short Sleep Duration and Obesity in Children and Adults,” Sleep 31 (2008): 619–626. 18517032 10.1093/sleep/31.5.619 PMC2398753

[7] F. P. Cappuccio , L. D'Elia , P. Strazzullo , and M. A. Miller , “Quantity and Quality of Sleep and Incidence of Type 2 Diabetes: A Systematic Review and Meta‐Analysis,” Diabetes Care 33 (2010): 414–420. 19910503 10.2337/dc09-1124 PMC2809295

[8] K. J. Egan , M. von Schantz , A. B. Negrao , et al., “Cohort Profile: The Baependi Heart Study‐A Family‐Based, Highly Admixed Cohort Study in a Rural Brazilian Town,” BMJ Open 6 (2016): e011598. 10.1136/bmjopen-2016-011598 PMC5093390 27797990

[9] R. B. Berry , C. L. Albertario , S. M. Harding , et al., The AASM Manual for the Scoring of Sleep and Associated Events: Rules, Terminology and Technical Specifications (Version 2.5) (American Academy of Sleep Medicine, 2018).

[10] M. E. Piche , A. Tchernof , and J. P. Despres , “Obesity Phenotypes, Diabetes, and Cardiovascular Diseases,” Circulation Research 126 (2020): 1477–1500. 32437302 10.1161/CIRCRESAHA.120.316101

[11] D. R. Matthews , J. P. Hosker , A. S. Rudenski , B. A. Naylor , D. F. Treacher , and R. C. Turner , “Homeostasis Model Assessment: Insulin Resistance and Beta‐Cell Function From Fasting Plasma Glucose and Insulin Concentrations in Man,” Diabetologia 28 (1985): 412–419. 3899825 10.1007/BF00280883

[12] T. Young , E. Shahar , F. J. Nieto , et al., “Predictors of Sleep‐Disordered Breathing in Community‐Dwelling Adults: The Sleep Heart Health Study,” Archives of Internal Medicine 162 (2002): 893–900. 11966340 10.1001/archinte.162.8.893

[13] M. F. Naufel , C. Frange , M. L. Andersen , et al., “Association Between Obesity and Sleep Disorders in Postmenopausal Women,” Menopause 25 (2018): 139–144. 28926516 10.1097/GME.0000000000000962

[14] B. Zhao , S. Sun , X. He , J. Yang , X. Ma , and B. Yan , “Sleep Fragmentation and the Risk of Obesity: The Sleep Heart Health Study,” Obesity (Silver Spring) 29 (2021): 1387–1393. 34196121 10.1002/oby.23193

[15] J. Theorell‐Haglow , C. Berne , C. Janson , C. Sahlin , and E. Lindberg , “Associations Between Short Sleep Duration and Central Obesity in Women,” Sleep 33 (2010): 593–598. 20469801 PMC2864874

[16] E. Tasali , R. Leproult , D. A. Ehrmann , and E. Van Cauter , “Slow‐Wave Sleep and the Risk of Type 2 Diabetes in Humans,” Proceedings of the National Academy of Sciences of the United States of America 105 (2008): 1044–1049. 18172212 10.1073/pnas.0706446105 PMC2242689

[17] Y. V. Ukraintseva , K. M. Liaukovich , K. A. Saltykov , D. A. Belov , and c. A. C. Nizhnik , “Selective Slow‐Wave Sleep Suppression Affects Glucose Tolerance and Melatonin Secretion. The Role of Sleep Architecture,” Sleep Medicine 67 (2020): 171–183. 31935619 10.1016/j.sleep.2019.11.1254

[18] N. Herzog , K. Jauch‐Chara , F. Hyzy , et al., “Selective Slow Wave Sleep but Not Rapid Eye Movement Sleep Suppression Impairs Morning Glucose Tolerance in Healthy Men,” Psychoneuroendocrinology 38 (2013): 2075–2082. 23602132 10.1016/j.psyneuen.2013.03.018

[19] B. Yan , B. Zhao , Y. Fan , et al., “The Association Between Sleep Efficiency and Diabetes Mellitus in Community‐Dwelling Individuals With or Without Sleep‐Disordered Breathing,” Journal of Diabetes 12 (2020): 215–223. 31503406 10.1111/1753-0407.12987

