# Supplementary material for: BMI, Sleep Architecture, and Glucose Metabolism: Insights From the Baependi Heart Study
Source: Obesity (Silver Spring). 2025 Jul 30;33(10):1849–54. doi: 10.1002/oby.24359 (PMC12404664; doi:10.1002/oby.24359)
Supplement: Supplementary file 1 — Figure S1. Flowchart detailing exclusion criteria and sample size. Figure S2. Histogram of the distribution of BMI in this sample. Figure S3. Histograms for the distribution of TST and REM by BMI group in this sample. Table S1. Adjusted comparisons of PSG sleep measures by BMI and AHI group. [file OBY-33-1849-s001.docx]

**Supplemental Material for:**

**Obesity, Sleep Architecture and Glucose Metabolism: Insights from the Baependi Heart Study**

Carolina Mendes Pessoa, MD, Tâmara P. Taporoski, PhD, Felipe Beijamini, PhD, Shaina J. Alexandria, PhD, Jose E. Krieger, MD, PhD, Malcolm von Schantz, PhD, Alexandre C. Pereira, MD, PhD, Kristen L. Knutson, PhD

This document contains the following:

Supplemental Figure S1. Flowchart detailing exclusion criteria and sample size.

Supplemental Figure S2. Histogram of the distribution of BMI in this sample.

Supplemental Figure S3. Histograms for the distribution of TST and REM by BMI group in this sample.

Supplemental Table S1. Adjusted comparisons of PSG sleep Measures by BMI and AHI group.

**Supplemental Figure S1.** Flowchart detailing exclusion criteria and sample size.

**Supplemental Figure S2.** Histogram of the distribution of BMI in this sample.

**Supplemental Figure S3.** Histograms for the distribution of TST (left panel) and REM (right panel) by BMI group in this sample.


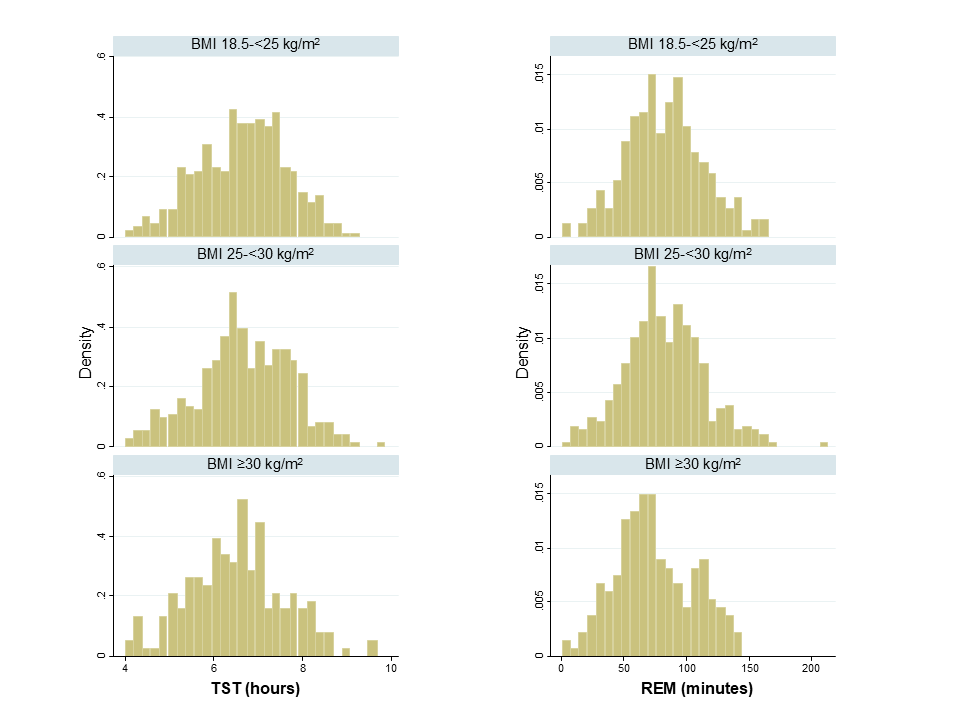


**Supplemental Table S1**. Adjusted comparisons of PSG sleep Measures by BMI and AHI group.^a^

|  | AHI<15 events/hour | | | AHI≥15 events/hour | | |
| --- | --- | --- | --- | --- | --- | --- |
|  | BMI 18.5<25 kg/m^2^ | BMI 25-<30 kg/m^2^ | BMI>30 kg/m^2^ | BMI 18.5<25 kg/m^2^ | BMI 25-<30 kg/m^2^ | BMI≥30 kg/m^2^ |
| N | 443 | 376 | 195 | 75 | 127 | 116 |
|  |  | B (95% CI) | B (95% CI) | B (95% CI) | B (95% CI) | B (95% CI) |
| TST (h) | referent | 0.06  (-0.09, 0.20)  p=.45 | -0.11  (-0.28, 0.07)  p=.24 | -.02  (-0.29, 0.24)  p=.85 | -0.12  (-0.33, 0.09)  p=.28 | **-0.22  (-0.44, -0.01) ^‡^**  **p=.04** |
| N2 (min) | referent | 3.2  (-4.2, 10.6)  p=.40 | 2.3 (-6.8, 11.4)  p=.62 | -5.2  (-18.6, 8.3)  p=.45 | -2.8  (-13.6, 8.0)  p=.62 | -9.1  (-20.2, 1.9)  p=.11 |
| N3 (min) | referent | -1.5  (-5.9, 2.9)  p=.50 | -0.1  (-5.3, 5.4)  p=.98 | **-12.5  (-20.5, -4.5) ^‡^**  **p=.002** | **-12.8  (-19.2, -6.3) ^‡^**  **p<.001** | **-18.3  (-24.8, -11.7) ^‡^**  **p<.001** |
| REM (min) | referent | 0.4  (-3.9, 4.7)  p=.86 | **-1.0 (-12.1, -1.8) ^‡^**  **p=.009** | -5.5  (-13.2, 2.3)  p=.17 | -3.2  (-9.4, 3.1)  p=.32 | **-7.4  (-13.8, -1.0) ^‡^**  **p=.023** |
| N2 (%) | referent | 0.2  (-1.1, 1.5)  p=.73 | 1.4  (-0.2, 3.0)  p=.10 | -1.1 (-3.5, 1.3)  p=.37 | -0.1  (-2.0, 1.8)  p=.93 | -0.8  (-2.8, 1.1)  p=.41 |
| N3 (%) | referent | -0.3 (-1.4, 0.8)  p=.62 | 0.3 (-1.1, 1.7)  p=.69 | **-3.0 (-5.1, -1.0) ^‡^**  **p=.004** | **-2.8 (-4.5, -1.2) ^‡^**  **p=.001** | **-4.1 (-5.8, -2.4) ^‡^**  **p<.001** |
| REM (%) | referent | -0.1 (-1.1, 0.8)  p=.77 | **-1.4 (-2.5, -0.2) ^‡^**  **p=.02** | -1.6  (-3.3, 0.1)  p=.06 | -0.6 (-1.9, 0.8)  p=.41 | -1.3  (-2.7, 0.1)  p=.07 |
| WASO (min) | referent | 2.1  (-3.4, 7.6)  p=.45 | 3.4 (-3.3, 10.2)  p=.32 | **17.4  (7.4, 27.4) ^‡^**  **p=.001** | **8.2  (0.2, 16.3)^‡^**  **p=.046** | **18.1 (9.8, 26.3) ^‡^**  **p<.001** |

^a^ Adjusted for age and gender

B = unstandardized regression coefficient; CI: confidence interval

^‡^ Adjusted p value<.05 compared to BMI<25
